# COVID-19: Military Nurses Leading Innovation Through Research, Clinical Care, Education, and Collaboration

**DOI:** 10.1093/milmed/usab009

**Published:** 2021-09-01

**Authors:** Heather C King, Laura A Talbot

**Affiliations:** TriService Nursing Research Program, Uniform Services University of the Health Sciences, Bethesda, MD 20814, USA; Department of Neurology, College of Medicine, University of Tennessee Health Science Center, Memphis, TN 38163, USA

## Abstract

Military nurses have been placed in the forefront of clinical and leadership roles during the COVID-19 pandemic. Serving in critical roles, military nurses have spearheaded innovations in clinical practice, conducted research, and implemented evidence-based practice projects that have advanced the capabilities of the Armed Forces Nurse Corps. This collection captures and highlights many of these military nursing contributions combating the COVID-19 pandemic.

## INTRODUCTION

The coronavirus pandemic has placed nurses from the Army Nurse Corps, Navy Nurse Corps, and Air Force Nurse Corps in the spotlight as essential members of the military healthcare team. Although every member of the healthcare team is important, the role of military nursing is unique. The COVID-19 pandemic has provided military nurses unprecedented opportunities to display their mission readiness in a rapidly evolving environment across the continuum of care. Innovations in clinical practices, implementing the best available science, continual education on evolving developments, and creating new and enduring collaborations have all contributed to readiness during this extraordinary demand for nursing care.

Nurses are known for their important clinical position at the bedside, but they are also demonstrating their unique ability to quickly identify patient care priorities, manage crises, improve healthcare environments, and provide exceptional leadership decisions and guidance during the current pandemic. With each new challenge during COVID-19, military nurses have responded with clinical solutions to provide safe, quality health care to our service members and their beneficiaries. The challenges of this pandemic include nursing staffing shortages, limited personal protective equipment, extended personal protective equipment wear times, rapidly evolving clinical knowledge and clinical care policies, staff quarantines, deployments, mental health concerns, telehealth, and virtual communications with patients, family members, and the healthcare team. As always, nurses meet these challenges while maintaining constant flexibility and adaptability during uncertain circumstances. The response of military nurses has been extraordinary and led to the supplement highlighting some of their most important contributions.

### Military Nurses: Top Clinicians and Scientists

Military nurses comprise one of the largest percentages of military healthcare personnel and one of the most deployed military specialties. These highly educated professionals frequently hold advanced academic degrees and professional certifications (Table I).^1^ One of the most important distinctions military nurses hold is their dual roles as clinical nurses and as commissioned officers in the Armed Forces. While military nurses’ role within the Defense Health Agency healthcare systems is similar to civilian clinical practice, their operational practice environments are unique and diverse—from field and surgical hospitals in operational theaters, to hospital ships serving global health engagement missions abroad, to large populous healthcare systems in the United States. These clinical practice settings span the globe and provide a unique perspective that encompasses diseases and scenarios that are uncommon in the United States, but prevalent in worldwide locations. Military nurses make tremendous sacrifices on behalf of our country to care for our nation’s wounded, injured, and sick service members. Military nurses are one of our country’s greatest assets as they serve the nation anywhere, anyplace, any time.

**TABLE I.** Armed Forces Nurse Corps Educational Levels FY20

Navy Nurse Corps		
Active duty component = 2,825 Commissioned Nurse Corps Officers		
Educational level	*n*	%
Bachelor of Science in Nursing	1,926	68%
Master’s degree	696	25%
Doctoral degree	203	7%
Reserve component = 1,310 Commissioned Nurse Corps Officers		
Educational data unavailable for reserve component		
Army Nurse Corps		
Active duty component = 3,224 Commissioned Nurse Corps Officers		
Educational level	*n*	%
Bachelor of Science in Nursing	1,968	61%
Master’s degree	902	28%
Doctoral degree	354	11%
Army Nurse Corps		
Reserve component = 4,084 Commissioned Nurse Corps Officers		
Educational level	*n*	%
Bachelor of Science in Nursing	2,741	67%
Master’s degree	1,081	26.6%
Doctoral degree	262	6.5%
Air Force Nurse Corps		
Active duty component = 3,225 Commissioned Nurse Corps Officers		
Educational level	*n*	%
Bachelor of Science in Nursing	1,976	61%
Master’s degree	1,016	32%
Doctoral degree	233	7%
Air Force Nurse Corps		
Reserve component = 1,352 Commissioned Nurse Corps Officers		
Educational level	*n*	%
Bachelor of Science in Nursing	859	65%
Master’s degree	425	31%
Doctoral degree	68	5%

One military nursing specialty is nurse scientist, professionals who bring their education and training in scientific writing and data analysis skills to mentor clinical nurses and encourage the dissemination of their important contributions to a wider audience. Though a small core group, military nurse scientists are prolific, productive, and essential to bringing research into military nursing practice settings. Military nurse scientists expand scientific knowledge related to the nursing care military nurses provide in austere and operation environments, but also inform military requirements, threats, and manning, identify new capabilities, and contribute to important healthcare policies. They also provide scientific mentorship to military nurses—to look for the best available science, to find gaps in military nursing care, to utilize data for clinical and leadership decisions, to use psychometrically sound instruments to capture meaningful data, and to implement evidence into all clinical settings.^2,3^ They challenge nurses to advance the scholarship of their practice. Recently, military nurse scientists have also worked tirelessly with nurses of all specialties, ranks, and services to advance evidence-based care in the Military Health System. The combined efforts of advancing research and evidence-based practice in the Military Health System are powerful tools to address the COVID-19 response and future health crises.

### COVID-19: Military Nursing Response

The current DoD’s priorities for COVID-19 are to maintain mission readiness, support interagency government endeavors to address the pandemic, and protect the troops, DoD’s civilian employees and contractors, and their families.^4^ Military nurses have contributed to the DoD’s COVID-19 priorities both at MTF locations and at civilian hospitals throughout the United States (Table I). The contributions of military nurses in these operational settings have provided invaluable clinical care. In addition to the required after-action reports, disseminating and publishing the contributions of military nurses in peer-reviewed journals is important. This supplement brought together nurses serving in clinical and leadership roles on the front lines of COVID-19 with nurse scientists serving as scholarly mentors to facilitate capturing these clinical experiences and lessons learned. This collaboration enhances the capability to disseminate innovations in nursing practice and optimally prepare for future crises. Nurses serving in primarily clinical and leadership roles bring a fresh perspective and ability to view gaps in military healthcare systems and military nursing practice and ask hard questions that are important. Military nurse scientists bring their education and training in scientific writing and data analysis skills to provide mentorship to nurses to encourage the dissemination of their important contributions to a wider audience. Additionally, this supplement serves to disseminate a variety of experiences during COVID-19: first-hand accounts, practice innovations, collaborations, case reports, educational efforts, research, evidence-based practice projects, commentaries, and tactical lessons learned from COVID-19 by military nurses.

### Relevant Research and Evidence-Based Practice for Future Pandemic Threats

COVID-19 has brought new awareness on the impact of worldwide pandemics and the need for future research to generate essential knowledge that will enhance the readiness of the Armed Forces. Future areas of research include

1. Current and emerging issues with direct application to military nursing care during wartime, peacetime, and humanitarian and national response.
2. Current and emerging issues related to healthcare readiness of individual service member and the military nurse force readiness.
3. Diversity and health disparities of service members as related to the military and military nursing.
4. Expanding research collaborations with multidisciplinary teams to address gaps in pandemic response and readiness.

Equally important is the ability to effectively implement evidence-based nursing care when limited effective treatment options are available for new and emerging health threats. Because of the unique clinical position of nurses, they are optimally positioned to readily define, implement, and evaluate the best available practices that advance quality and safety in military health care. Areas important to address include

1. Evidence-based practice projects that define, implement, and evaluate infection prevention and control practices during wartime, peacetime, and humanitarian and national response.
2. Evidence-based practice projects that define, implement, and evaluate communication tools, such as telehealth technology, to expand access to healthcare services for military service members and their families during the COVID-19 pandemic and beyond.
3. Evidence-based practice projects that assess readiness, gather evidence, set up training, promote staff safety, and bolster peer support to strengthen the military healthcare system.
4. Evidence-based practice projects that assess healthcare provider risk (both physical and mental), reduce harm, and apply workplace safety interventions and evaluate outcomes during COVID-19 and other emerging pandemic threats.
5. Evidence-based practice projects that define, implement, and evaluate leadership decisions, leveraging data analytics to drive time-sensitive decisions to improve patient outcomes in military health care.

Empowering military nurses in these areas is the key to reducing mortality and improving patient outcomes across the continuum of care.

### TriService Nursing Research Program

The TriService Nursing Research Program (TSNRP) provides modest, but needed, infrastructure support for military nurses to conduct research and to collaborate across the Armed Services. Building professional relationships is a hallmark of the TSNRP and is evidenced by the collaboration between the frontline clinicians and military nurse scientists who created this supplement.

These identified research gaps and evidence-based implementation areas align with the TSNRP’s long-standing funding priorities of (1) force health protection, (2) nursing competencies and practice, and (3) leadership, ethics, and mentoring.

This supplement is also aligned with the TSNRP’s strategic goal of developing and strengthening the community of nurse scholars to generate new knowledge and translate it into practice.

It is hoped that this experience will serve as a future model to continue to develop the scholarship of military nurses and encourage the next generation of military nurses to disseminate important military nursing contributions through professional publication. Undeniably, the greatest strength of the TSNRP community are the military nurses—passionate, determined, resolute, and laser focused on readiness.

Ironically, the COVID-19 pandemic occurred during the International Year of the Nurse as declared by the World Health Organization. It seems appropriate that in 2020 military nurses continued the legacy of advancing nursing scholarship to advance the care our service members and beneficiaries receive. Working with this dedicated group of nurses has been an incredible experience and truly contributes to paving the way for advancing military nursing.
